# Improving Healthy Food Choices in Low-Income Settings in the United States Using Behavioral Economic-Based Adaptations to Choice Architecture

**DOI:** 10.3389/fnut.2021.734991

**Published:** 2021-10-06

**Authors:** Emma Anderson, Ruobin Wei, Binkai Liu, Rachel Plummer, Heather Kelahan, Martha Tamez, Abrania Marrero, Shilpa Bhupathiraju, Josiemer Mattei

**Affiliations:** ^1^Department of Nutrition, Harvard T. H. Chan School of Public Health, Boston, MA, United States; ^2^Department of Epidemiology, Harvard T. H. Chan School of Public Health, Boston, MA, United States

**Keywords:** behavioral economics, choice architecture, food choices, low-income populations, food pantries, SNAP retailers

## Abstract

Low diet quality is a significant public health problem in the United States, especially among low-income populations. The food environment influences dietary choices. When applied to eating behavior, behavioral economics (BE) recognizes that decision biases instigated by a food environment saturated with unhealthy foods may lead people to purchase such foods, even when they possess the necessary information and skills to make healthy dietary choices. Choice architecture, a BE concept that involves modifying the appeal or availability of choices to “nudge” people toward a certain choice, retains freedom of choice but makes unhealthy options less convenient or visible. Choice architecture has been demonstrated to influence food choices in various settings, including supermarkets, convenience stores, and food pantries. These modifications are low-cost and feasible to implement, making them a viable strategy to help “nudge” patrons toward healthier choices in food establishments serving low-income populations, including food pantries and retailers accepting the Supplemental Nutrition Assistance Program. This narrative review searched, appraised, and underscored the strengths and limitations of extant research studies that used choice architecture adaptations to influence food choices among low-income populations in the United States. Findings from studies in food pantry settings suggest the potential of BE strategies to improve the healthfulness of food choices and dietary intake in low-income populations. In food retail settings, research suggests that BE strategies increase sales of healthy foods, like fruits and vegetables. We identify new areas of research needed to determine if BE-based modifications in low-income settings have sustained impacts on diet quality.

## Introduction

Low diet quality represents a significant public health problem in the United States (US). Estimates state that 82% of US adults aged 20–49 years and 73% of adults over 50 years score poorly on measures of diet (1). Low-income populations are disproportionately impacted by low dietary quality, as evidence suggests that compared to high-income households, low-income households purchase less healthful foods and have significantly lower dietary quality (2, 3). Low diet quality is associated with an elevated risk of many health conditions, including cancer, type 2 diabetes, cardiovascular disease, and overall mortality (4, 5). Dietary choices are largely shaped by the food environment, or the availability, affordability, and convenience of food (6). In retail settings such as supermarkets, grocery stores, and convenience stores, efforts informed by behavioral economics are currently underway to leverage the food environments at food retailers to promote and enable healthy dietary choices (7–11).

Theory in behavioral economics recognizes that even when people possess the necessary information and skills, decision biases and other pitfalls may lead them to make decisions that are not in their best interest (7). Behavioral economics can offer insight into the decision-making processes involved in making health-promoting dietary choices (7, 8). Choice architecture, a behavioral economics concept that involves modifying the appeal or availability of choices in an environment to “nudge” people toward a particular choice, has been used to influence a wide range of health behaviors, including dietary choices (8). Examples of changes to choice architecture include changing store layouts to make healthy foods more accessible, grouping food to showcase healthy options, and providing quick, easy-to-understand information. Evidence suggests that strategic manipulation of the food environment has the potential to influence food choices in a variety of environments, including grocery stores, convenience stores, and food pantries (8–11). A “CAN” approach in nutrition interventions has also been suggested, which aims to make healthier food choices more Convenient, Attractive, and Normal to select (12).

Access to nutritious food is considered an essential social determinant of health due to the direct impact of nutrition on health (13). An estimated 10.5% of U.S. households, or 35 million individuals, were food insecure during 2019, indicating that they lacked access or funds to purchase enough healthy and safe food for all household members (14). To help alleviate this burden, low-income individuals in the US often access food pantries or apply for the Supplemental Nutrition Assistance Program (SNAP), a government initiative that provides funds to income-qualifying individuals and families for food purchases at eligible vendors (15). The focus of these programs is to increase food accessibility in food insecure populations; thus, improving the diet quality of low-income populations has not been a priority historically (16, 17). Given concerns of low diet quality among low-income populations, there is a substantial need for interventions that facilitate improved food choices in these settings. Implementing behavioral economics strategies in SNAP-approved retailers and other free or low-cost food sources in the US may be one such solution.

This mini-review summarizes the strengths and limitations of behavioral economics strategies, specifically choice architecture adaptations, to improve the quality of foods chosen by low-income populations in food retail settings and pantries. We searched PubMed using terms such as “behavioral economics,” “choice architecture,” “nudging,” “food choices,” “diet quality,” “food pantries,” and “SNAP.” Although behavioral economics strategies have been examined in health promotion and healthcare contexts (7), and choice architecture approaches to promote healthy eating have been studied in retail settings among various populations (18, 19), this review distinctly focused on choice architecture applications in food environments serving low-income populations in the US, including food pantries and SNAP-authorized retailers. Evidence from a limited body of research conducted in food retail and food pantry settings is presented to gain an understanding of the effect of choice architecture approaches on the food choices of low-income populations in the US.

### Diet Quality of Low-Income Populations

Research suggests that low-income households tend to purchase and consume foods that are less healthy than higher-income households (2, 16, 17). Compared to high-income households, food purchases of low-income households had lower diet quality scores based upon adherence to the United States Department of Agriculture's (USDA) dietary guidelines (2, 3). Average scores of food purchases among SNAP-participating households also tend to be lower than non-participants regardless of income (3, 17). Food pantry clients demonstrate similarly low dietary quality, attributable to lower than recommended intake of fruit, vegetables, and whole grains and higher than recommended intake of fatty meats, refined grains, and sugary beverages (20).

The mechanisms driving unhealthy dietary choices among low-income populations are complex and not limited to individual choices. A disproportionately higher presence of marketing for unhealthy foods in low-income communities is a main contributor (21). For example, a study conducted in California found a higher density of unhealthy food and beverage advertisements in low-income neighborhoods compared with higher-income neighborhoods, with the highest density in Latino and African-American communities (22). Another study in SNAP-authorized retailers in New York state revealed that in-store marketing and display of sugary beverages doubled during SNAP benefit issuance days compared with other days of the month (23). In neighborhoods with high SNAP enrollment, the odds of a retailer having sugar-sweetened beverage displays were also more than four times higher during SNAP benefit issuance days compared with non-issuance days; there were no differences in marketing for low-calorie or unsweetened beverages (23). The combination of targeted advertising for unhealthy foods in low-income populations, along with financial constraints often faced by low-income households, negatively impacts the diet quality of low-income populations.

### The Impact of SNAP

SNAP serves over 42 million individuals in the US and provides a major source of nutrition assistance for low-income populations. Most SNAP-approved vendors are small grocery and convenience stores (78.0% of SNAP vendors); however, the majority of benefits are redeemed at superstores and supermarkets (82.5% of SNAP redemptions), which together make up 15.4% of SNAP retailers (24, 25). While SNAP does reduce food insecurity, the program does little to promote diet quality (26, 27). There are few restrictions on what can be purchased with SNAP, as benefits can be used on most food items, except immediately consumable prepared foods, alcohol, vitamins, supplements, or live animals (15). Given limited restrictions, changing choice architecture to nudge consumers toward healthier choices may be a well-suited strategy to improve dietary intake and minimize individual intervention or paternalism (28). Implementing choice architecture interventions in SNAP-approved retail settings has the potential to improve the impact of federal food assistance programs on nutrition.

## Evidence of Behavioral Economics on Food Choices

Intervention studies evaluating the effects of changes to choice architecture on consumer food choices have been conducted in various settings, including food pantries, convenience stores, and supermarkets. These interventions have demonstrated the potential to nudge people to make healthier food choices in food pantries and retailers in underserved, low-income communities (Table 1).

**Table 1:** Intervention studies and programs applying choice architecture modifications to encourage healthier food choices in food pantry and retail settings for low-income populations.

**Study design and setting**	**Intervention**	**Findings**	**References**
**Food pantries**			
Experimental study testing an intervention (SuperShelf) implemented in pantries in Minnesota	First phase of the intervention increased quantities and varieties of healthy options in the food pantry. In the second phase, which was informed by BE, food was sorted by food groups, fruits and vegetables were displayed in colorful bins, and healthy items were placed at eye level on shelves. Two food pantries received intervention. Two other pantries did not (control). Pantry selection was not randomized. Patrons were surveyed at baseline and 4-month (end of intervention).	Eight and 19-point increase in 2 pantries with intervention, respectively, in total healthy eating index (HEI-2010) compared to baseline. One pantry had a significant 12 point (HEI-2010) increase in nutritional quality of foods selected (*p* < 0.0001).	Caspi et al. (29)
Evaluation of a nudge program (Thumbs Up) in urban food pantries in Utah	Six pantries that had partnered with the program for at least 4 months were non-randomly selected into the study and surveys were collected from patrons. Highly visible shelf labels with colorful “thumbs up” images that said “Healthy Choice” in English or Spanish were placed under targeted healthy foods, and bilingual posters were displayed in the pantry to raise awareness about the shelf tags and target foods.	Client questionnaires showed that 94% thought the program facilitated making healthy food choices, and over two-thirds reported that shelf labels encouraged them to choose originally unfamiliar healthy foods. Moreover, nearly 70% of those surveyed reported that their family ate healthier after these nudges were introduced in the pantry.	Coombs et al. (10)
Randomized control trial in a New York City food pantry	Changing choice architecture by changing the shelf placement order (front vs. back) and packaging (boxed vs. unboxed) of healthy food to nudge clients toward healthy choices. The study displayed an assortment of healthier protein bars along with traditional desserts at the pantries' dessert table. Placement order and packaging interventions were paired and 443 patrons were randomized to one of the following paired arms: “Front-Boxed,” “Front-Unboxed,” “Back-Unboxed,” and “Back-Boxed.” The interventions took place over four successive days of operation.	The study displayed an assortment of healthier protein bars along with traditional desserts at the pantries' dessert table. They found that the odds of selecting a protein bar instead of desserts at the front of the pantry was 1.69 (95% CI: 1.09, 2.52), compared to those at the back. The odds of selecting a protein bar presented in its original box was 1.92 (95% CI: 1.24, 2.99) compared to unboxed.	Wilson et al. (11)
**Food retailers**			
Two rural counties in central North Carolina, USA.	Transaction data from a pair of grocery stores and a pair of convenience stores were analyzed to study the association between “nudges” and sales of promoted items. Stores in each pair were randomized to either receive interventions or to serve as the control. During a 22-week period, three nudges were conducted individually and together only in intervention stores for 4 weeks, separated by 2 weeks of washout period. Nudges included: a “cognitive fatigue” experiment (floor arrows to produce sections); a “scarcity” experiment (having a “limited amount” message on a sign in the produce section); and a “product placement” experiment (granola bars moved to candy aisle).	In grocery stores, neither individual nor combined nudges resulted in significant differences in the sales of the promoted items during the invention time frame. In convenience store settings, stores implementing all three nudges simultaneously had a 25% higher sales increase compared to control stores.	Chapman et al. (9)
Evaluation of the Healthy Bodegas Initiative implemented in New York City corner stores	Sixty corner stores (bodegas) in several low-income neighborhoods were recruited. Stores were advised to implement changes such as placing bottled water at eye level, adding posters to promote healthy food items (e.g., low-sodium canned foods), and increasing the stocking of healthy foods (e.g., increase the variety of fruits and vegetables displayed). Owners from 55 stores and 617 customers from a subset of stores were surveyed before and after the interventions.	78% of store owners reported increased sales of healthier foods. Among customers, the proportion buying 1 or more bottles of water improved from 6 to 12%. Customers who purchased promoted healthy items increased from 5 to 16%.	Dannefer et al. (30)
Cluster randomized controlled trial conducted in 8 urban supermarkets in low-income neighborhoods	Four supermarkets received the intervention, 4 did not (control). The intervention consisted of 6 months of in-store marketing strategies. These strategies included increasing the number of facings (visible shelf slots) for targeted healthy products, placing the healthy products in the most prominent locations, signage advertising the healthy, making end caps and dead spaces healthier, and other strategies as applicable.	During the same time period, sales of skim milk, 1% milk, water, and 2 of 3 targeted frozen meals were significantly greater in intervention stores than control stores. Sales of cereal, whole milk, 2% milk, beverages, and diet beverages did not differ between stores.	Foster et al. (31)
Store managers at six corner stores in low-income neighborhoods in Chelsea, MA that serve adult WIC participants	Six corner stores in Chelsea, MA were enrolled (three randomized to intervention and three to control). The 5-month intervention aimed to increase both the visibility and quality of the store's fresh produce. Interventions were tailored to the individual needs of the store, and stores were provided with materials to improve their produce displays, including new shelving, baskets, etc. Produce consultant advised owners about strategies for stocking and maintaining high-quality produce. Study staff helped store owners make produce displays more immediately visible and attractive, placing them at the front of the store. No changes were made at control stores.	During the intervention, fruit and vegetable sales at stores receiving the intervention significantly increased by $40/month and decreased by $23/month in control stores (*p* = 0.036). No other purchases changed. WIC customers who shopped at intervention stores reported that they had increased their fruit and vegetable purchases more than WIC customers at control stores (18 vs. −2%), but this was not statistically significant (*p* = 0.11).	Thorndike et al. (32)
Ten corner stores in Baltimore City, MD that serve adult WIC participants	Eight corner stores were intervention sites and two were control sites. There are four stages of intervention, and each stage contains a 1-month treatment period and a 1-month no-treatment period. In the first stage, 4 treatments were randomized to eight intervention stores (2 each), and stores received one more treatment in the next stage until all 4 treatments were implemented. The treatments included store owner training, point of purchase promotion, product placement, and product grouping. No changes were made to the 2 control sites.	All 4 strategies studied were deemed feasible and had high reach and adherence. The store owner training strategy was the most successful and produced positive changes in stocking of WIC foods, total sales of WIC foods, and WIC purchases. WIC purchases of infant food, fruits and vegetables, and grains were positively correlated with numbers of BE strategies implemented.	Wensel et al., (33)

### Food Pantries

Feeding America's network of 58,000 food pantries serves ~46.5 million individuals in the US every year (34). Although being low-income is not a criterion for participating in food pantries, food pantry clients are generally low-income (20). Implementing low-cost and unobtrusive changes to choice architecture at food pantries has demonstrated the potential to nudge low-income patrons to make healthier food choices. A randomized controlled trial in New York food pantries evaluated the effectiveness of changes to healthy food options' shelf placement order (i.e., making products easier to see and grab) and packaging (i.e., making products more visually appealing) (11). The study displayed an assortment of healthier protein bars and traditional desserts at the pantries' dessert table. They found that the odds of selecting a protein bar at the front of the pantry was 1.69 (95% CI: 1.09, 2.52) compared to those at the back. The odds of selecting a protein bar presented in its original box was 1.92 (95% CI: 1.24, 2.99) compared to those unboxed. These nudges successfully applied the convenience and attractiveness aspects of the “CAN” approach to increase patrons' selection of targeted foods. Another study evaluated changing shelf signage and posters in urban food pantries in Utah (10). Highly visible shelf labels with colorful “thumbs up” images that said “Healthy Choice” in English or Spanish were placed under targeted healthy foods, and bilingual posters were displayed in the pantry to raise awareness about the shelf tags and target foods. After these changes, 94% of surveyed patrons thought the program facilitated choosing healthy foods, and over two-thirds reported that shelf labels encouraged them to choose originally unfamiliar healthy foods. Nearly 70% of those surveyed reported that their family ate healthier after these nudges were introduced in the pantry. Multiple exposures to targeted healthy foods—including hanging pictures of fruit and vegetables (35), displaying fruits and vegetables in colorful bins, and placing healthy items at eye level on shelves (29)—nudge pantry patrons toward healthier options. Many of these simple, unobtrusive nudges are easy and inexpensive, making them appropriate for settings with limited resources (11).

Despite promising findings, the current evidence base is limited, including a lack of randomized trials and longitudinal studies examining the long-term effects of nudges on food pantry patrons' food choices (36). Also, these studies tend to rely heavily on self-reported and observational data, thus, more standardized and objective measurements of changes in diet quality in low-income food pantry patrons are also needed (20). Additionally, given potential differences in food choices attributable to cultural preferences (10), future research is needed to better understand how cultural appropriateness can be incorporated into choice architecture across diverse populations.

### SNAP Retailers

Changes to choice architecture have been shown to promote sales of healthy foods in retail stores that accept SNAP redemption, often located in low-income communities. A cluster-randomized controlled trial showed that increasing healthy product visibility and signage in supermarkets located in low-income neighborhoods, most of which were SNAP retailers, resulted in significantly greater sales of several targeted options (e.g., skim milk, 1% milk, water, selected frozen meals) compared to stores with no intervention within 6 months (31). Another intervention study in rural Southern US counties demonstrated that a combination of three changes to choice architecture (i.e., floor arrows to healthy foods, signage indicating healthy stock, and prominent healthy product placement) significantly increased sales of promoted nutritious foods in SNAP-participating convenience stores (9). Similarly, after increasing product visibility, variety, and availability of healthier foods in 55 corner stores located in several low-income neighborhoods in New York, 78% of store owners reported increased sales of healthier foods (30). While not conducted in low-income settings, another field experiment found that displaying bundles of healthy foods at grocery stores increased purchases of fruit and vegetables by 15% with a small price discount and 9% without any price discount (37). Along with interventions, some observational studies have similarly found placement strategies in food stores to be positively associated with higher sales of healthy foods (18, 38, 39).

The success of these choice architecture changes at supermarkets, convenience stores, corner stores, and grocery stores in nudging consumers to choose healthier options demonstrates potential in using these inexpensive strategies to promote sales of healthy foods at food retailers (18, 24, 25). However, limitations should be mentioned, including small sample sizes, potential self-reporting and recall bias, inconsistent marketing methods, potential loss of generalizability in other seasons, and price and inventory fluctuations (29, 36). Lack of randomization, short washout periods, and heterogeneity across studies (9), as well as highly controlled experimental conditions, may also threaten internal and external validity (37). Some studies also incorporated other promotional strategies outside of nudges [e.g., nutrition education (40), and taste testing (40, 41)], making it difficult to discern the specific effects of choice architecture strategies. Future studies investigating adaptations to choice architecture that target food choices of low-income populations should address these limitations (8).

### Online

The recent expansion of online ordering capabilities to SNAP retailers presents an opportunity to implement behavioral nudges online. The SNAP Online Purchasing Pilot, a program testing online grocery purchasing in SNAP, was launched in April 2019. By early 2021, online purchasing became available as an option for SNAP beneficiaries in 48 states at large nationwide retailers (42). A recent report revealed rapid uptake of SNAP Online Purchasing during 2020, which enabled participating households to adhere to COVID-19 social distancing recommendations while offering opportunities to safely access fresh, nutritious food (43). A randomized trial conducted in an online supermarket setting in the Netherlands examining whether nudges and pricing strategies increase purchases of healthy foods found that combining health-related price discounts with nudges stimulated purchases of healthy foods for both low and high-income populations (44). A secondary analysis of this virtual supermarket experiment revealed that nudging and pricing strategies had differential effects on purchases of different food groups, as purchases of healthy items from fruits and vegetables, grains, and dairy groups increased significantly, whereas protein and beverage purchases did not significantly change (45). Given the increased reliance on online grocery ordering, which increased nearly 300% during the COVID-19 pandemic (46), online ordering presents new opportunities for nudging consumers, including SNAP participants, toward healthy food purchases. Addressing structural barriers around digital literacy, technology ownership, and reliable internet access will be essential to conduct behavioral economics strategies in online food shopping among low-income populations.

## Discussion

In contrast to nutrition education or other dietary interventions that put the onus on the individual, behavioral economics strategies may reduce effort and improve the convenience of making healthier food choices. Choice architecture nudges individuals toward healthier options without restricting their choices by making certain options more convenient or visible (47, 48). Behavioral economics strategies also require little to no time commitment by the consumer. Time is often a barrier to participation in health promotion programs, especially among low-income groups (49); interventions that enhance individual nutrition knowledge can also be time-intensive and costly to implement. Although behavioral economic interventions also entail a cost (50, 51), subtle choice architecture modification such as positioning healthy products to eye level is more cost-effective compared to providing monetary incentives or formally designed education sessions (52). Thus, behavioral economics approaches provide a feasible and low-cost intervention that can be implemented in various settings, including food pantries and retailers with limited resources (19, 53).

Despite these advantages, there are potential shortcomings of using changes to choice architecture as a nutrition intervention. Individuals make food choices in various settings beyond retail food establishments, including at home, schools, and worksites. While behavioral nudges may influence behavior at the moment, in another environment without such nudges, people may continue to make unhealthy choices (53). In addition, approaches do not directly address structural barriers to healthy eating, such as lack of time and resources to prepare healthy food, access to retailers that offer a wide array of healthy foods, and access to safe and reliable transportation to healthy food retailers (19, 54, 55). More effective approaches that sustain behavior change over time should address multiple social determinants of health and alter the food environment to make it easier to access healthy foods (19). Future research should examine the optimal design for behavioral economics interventions or systemic changes among food retailers in low-income settings (8, 20, 32).

A challenge to implementing behavioral economics-based interventions in food retail settings is establishing partnerships with food retailers. At smaller stores, managers are most often responsible for the layout and selection of the products available at their stores (56). However, at larger stores, suppliers can pay for prime shelf real estate, and unhealthy products are more likely to be stocked by the supplier while stocking healthy options is often the manager's choice (56, 57). Other factors, such as consumer demand, retailers' knowledge regarding health promotion, views about choice architecture in food retail stores, and community demographics, may influence retailers' ability and willingness to use strategies that promote healthy consumer choices (56). Collectively, these findings suggest that it is important for interventions implemented in retail settings to engage store managers as stakeholders, in addition to aligning with their business models and resources at hand (56, 58). More research is needed to identify feasible, cost-effective, and acceptable changes to choice architecture capable of supporting healthy consumer choices (56, 59), as well as to understand how to incentivize retailers to take part in these changes (48).

Research evaluating the efficacy of interventions aimed at promoting healthy food choices in low-income settings using behavioral economics-informed approaches, most of which has been conducted in food pantry settings, suggests that such approaches may improve the healthfulness of food purchases and dietary intake in low-income populations. However, more research is needed to determine the optimal design of interventions that leverage behavioral economics in retailers serving low-income consumers (8, 20, 32). Prior to implementing such interventions, researchers must identify approaches that can be appropriately translated to these populations to support healthy food choices, with feasibility, cost-effectiveness, cultural competency, and acceptability as key considerations (59, 60). Also, given the demonstrated potential of behavioral economics-based approaches to improve food choices, policy initiatives encouraging alterations to food environments, such as SNAP-authorized retailers, informed by behavioral economics and aimed at promoting healthy food choices is a promising avenue to address poor diet quality (18, 19, 61). More research is needed across diverse food retail store contexts to determine intervention approaches appropriate for SNAP-authorized retailers that promote the purchase and consumption of healthy foods among low-income populations (18, 61).

## Conclusion

Low diet quality is a public health concern that disproportionately impacts low-income populations (17, 62). Because of the vast reach of SNAP and food pantries for low-income populations, both serve as optimal vectors for interventions to improve diet quality in this population. As they currently exist, SNAP and food pantries preserve individuals' freedom of choice, which is crucial to maintaining autonomy and agency for individuals. For this reason, behavioral economic approaches, like changes to choice architecture, are well-suited to be implemented in food pantries and SNAP vendors to make healthy choices easier for low-income individuals (19, 48). While limitations to the extant evidence and challenges to future studies remain, changes to choice architecture at locations like food pantries are likely to nudge low-income participants to make healthier choices. Thus, there is potential for changes to choice architecture in retail food settings to improve the diets of low-income populations and SNAP participants.

## Author Contributions

EA, RW, and BL conceptualized the topic, researched and analyzed the background literature, wrote the manuscript, and including interpretations. RP and HK researched and analyzed the background literature, wrote portions of the manuscript, and including interpretations. AM, MT, SB, and JM provided substantial scholarly guidance on the conception of the topic, manuscript draft and interpretation, and revised the manuscript critically for intellectual content. All the authors approved the final version of the manuscript, ensured the accuracy and integrity of the work, and agreed to be accountable for all aspects of the work.

## Conflict of Interest

The authors declare that the research was conducted in the absence of any commercial or financial relationships that could be construed as a potential conflict of interest.

## Publisher's Note

All claims expressed in this article are solely those of the authors and do not necessarily represent those of their affiliated organizations, or those of the publisher, the editors and the reviewers. Any product that may be evaluated in this article, or claim that may be made by its manufacturer, is not guaranteed or endorsed by the publisher.
